# Agaric in Night-Sweats of Phthisis

**Published:** 1883-01

**Authors:** 


AGARIC IN THE NIGHT-SWEATS OF PHTHISIS.

Agaric * is a fungus of the larch. It is known in botany as the agaricus laricis, boletus laricis, and the polyporous officinalis.

It is an old remedy, having been in use in the treatment of the phthisis for over a century. Dahen used it successfully in 1797. Rayer, of Paris, employed agaric in a large number of cases at the Hôpital de la Charité. A pamphlet was published by E. Bisson, in 1832, containing an account of the results of its use in fourteen cases, as follows: 1. White agaric may be employed with advantage in the treatment of the night sweating of phthisis. 2. In doses of four, six, eight, or ten grains, given at bedtime, for some days, it usually checks sweating, when the patient is not at the same time suffering from diarrhœa. 3. When diarrhœa is also a prominent symptom, the agaric should be given in combination with opium. 4. When the diarrhœa is persistent, and not checked by opium, agaric is contra-indicated. 5. Agaric not only checks sweating, but induces sleep and prevents exhaustion. 6. Even if powerless to cure phthisis, it retards the progress of the case, and relieves the patient of one of the most dangerous and distressing symptoms. Andral, in the Hôpital de la Pitié, used it extensively. The dose employed was usually from six to eight grains, in two pills, gradually increased to thirty-six grains, divided into six pills. On one occasion, he gave a patient first

* Dr. Murrell—*The Practitioner*, November, 1882.

thirty, then forty-eight, and finally sixty grains. It checked the sweating, but caused violent purging. Trousseau usually gave about nine grains at bedtime.*

Dr. Murrell has treated sixty-four cases of night sweating with agaric. In fifteen of these there was cavity of the lung. To ten of the patients he gave pills of three grains of the powder each. The sweating was checked, but the action was slow, and not certain.

The pills of the powder being bulky, he gave to eight patients three-grain pills of the extract of agaric. Each pill was the equivalent of nine grains of the powder. The results were better. To thirteen patients thirty-grain doses of the powder were given. This set up purging. Twenty-grain doses reduced the sweating, and did not cause diarrhœa.

Dr. Murrell concludes that it is a good remedy, and there are times when it may be used to advantage; but he much doubts if it is equal to atropia, picrotoxine, pilocarpine or Dover's powder.

Dr. John M. Young † takes a more favorable view of the drug. He used a tincture of the powder, of the strength of ten grains to the drachm, but the active principle agaricine, in 1-12th-grain doses, was found a more convenient mode of administration.

His conclusions are as follows: 1. Night sweating becomes lessened proportionately to the amount of the drug administered; and if sufficient doses be given, becomes effectually checked or prevented, according to the time of administration. It works well in cases of sweating not dependent on phthisis. In the quickness of its action it resembles atropin. 2. Its effect in sweating is not more marked than its effect in producing sound sleep, and relieving troublesome cough, especially that of phthisis. This is the fact most notable to the patients themselves, a gradually increasing feeling of drowsiness following its administration, in most cases. In one case in particular, the first large dose of tincture of agaricus checked troublesome and painful coughing during the night.

---

* Agaric has a place in the French Codex. Its preparations are a powder and a liquid extract. A general account is to be found in the *National Dispensatory of Stillé and Maisch.*

† *Braithwaite's Retrospect*, July, 1882, p. 72.

Fungus Agarici is mentioned in Quincy's "Compleat English Dispensatory," London, 1749. It is classed as an eccoprotic, and no mention is made of its being useful in any other way. Dr. Grant, of Ottawa, advocated its use in acute rheumatism. His article is to be found in the *British American Journal of Medical Science* for 1862, Vol. III., p. 92.

